# Correlation Between Thalamus-Related Functional Connectivity and Serum BDNF Levels During the Periovulatory Phase of Primary Dysmenorrhea

**DOI:** 10.3389/fnhum.2019.00333

**Published:** 2019-09-30

**Authors:** Fang Han, Hongjuan Liu, Ke Wang, Jing Yang, Ling Yang, Jixin Liu, Ming Zhang, Wanghuan Dun

**Affiliations:** ^1^Department of Rehabilitation Medicine, First Affiliated Hospital of Xi'an Jiaotong University, Xi'an, China; ^2^Department of Intensive Care Unit, First Affiliated Hospital of Xi'an Jiaotong University, Xi'an, China; ^3^Department of Medical Imaging, First Affiliated Hospital of Xi'an Jiaotong University, Xi'an, China; ^4^Department of Medical Imaging, Chong Qing Medical University, Chong Qing, China; ^5^School of Life Science and Technology, Xidian University, Xi'an, China

**Keywords:** primary dysmenorrhea, chronic pain, thalamus, neuroimaging, fMRI, menstrual pain

## Abstract

The thalamus is a key region for the transmission of nociceptive information in the central modulation of pain and has been studied in the setting of numerous chronic pain conditions. Brain-derived neurotrophic factor (BDNF) is considered an important modulator for mediating nociceptive pathways in chronic pain. The present study aimed to investigate whether there was thalamus-related abnormal functional connectivity or relevant serum BDNF level alterations during periovulation in long-term primary dysmenorrhea (PDM). Thalamic subregions were defined according to the Human Brainnetome Atlas. Functional connectivity analyses were performed in 36 patients in the periovulatory phase and 29 age-, education-, and gender-matched healthy controls. Serum BDNF levels were evaluated by enzyme-linked immunosorbent assay and a significantly higher BDNF level was detected in PDM patients. Compared with HCs, PDM patients had abnormal functional connectivity of thalamic-subregions, mainly involving with prefrontal cortex, sensorimotor cortex, and temporal cortex. In addition, the functional connectivity of thalamic-subregions showed significant interactive effect correlated with serum BDNF level between PDM and HCs. It has been suggested that there were maladaptive or adoptive alteration associated with chronic menstrual pain even without the ongoing menstrual pain. BDNF might play a role in the development and chronicity of central nervous system dysfunction. These findings provided more accurate information about the involvement of the thalamus in the pathophysiology of PDM.

## Introduction

Primary dysmenorrhea (PDM) is an abnormal cramping pain that manifests with the onset of menstruation in every cycle but without organic etiology, which may impact 20–90% of females and is considered to be the most common gynecological disorder in reproductive age (Dawood, 2006; Morrow and Naumburg, 2009). According to a recent review, hyperalgesia, and central sensitization within or outside the area of referred menstrual pain may be a risk factor for developing chronic pain (Iacovides et al., 2015). Maladaptive perception of noxious stimuli (Giamberardino et al., 1997; Vincent et al., 2011) and aberrant central pain pathway during periovulation (a trait-related property) has been suggested by a number of neuroimaging studies focusing on cerebral mechanisms (Tu et al., 2009, 2010; Wei et al., 2016a) in PDM. Significant correlations between altered white matter integrity, an abnormal cerebral pain pathway network during periovulation and the intensity of perceived menstrual pain was reported by us previously (Dun et al., 2017a,b); however, clear associations between trait-related pain perception pathway and properties of dysmenorrhea remain largely unclear.

Within ascending and descending pain pathways, the thalamus is the key site for the transmission of nociceptive information to and from the sensory cortex (Zhang et al., 1995; Gauriau and Bernard, 2004; EG, 2007a,b; Todd, 2010). During spatial or non-spatial discrimination of pain stimuli, intensity discrimination-related activation was detected in the medial thalamus, anterior insula and prefrontal cortex (Oshiro et al., 2009). Similar dynamic patterns of thalamic function were also reported in another experimental assessment of thermal pain in which the thalamus was identified as critical in the prediction of pain intensity (Wager et al., 2013). Additionally, it's evidenced by a rodent experiment that inhibition of thalamic sensory neurons was in part responsible for the motor cortex stimulation-induced antinociception (Pagano et al., 2012). These studies suggested that thalamic served important functions in pain processing, involving nociceptive perception, and modulation. However, thalamus is not only a passive relay station for sensory information, but also an integrative hub for brain signal processing between cortical cortex due to its extensive connections with the cerebral cortex (Hwang et al., 2017). For example, communication between the mediodorsal thalamus and the medial frontal cortex may modulate emotion and cognition (Kong et al., 2018), while elevated activity between the medial dorsal thalamus and anterior cingulate cortex may exacerbate pain-related aversion (Meda et al., 2019). Although previous studies have reported altered brain circuits in PDM to mostly be involved with pain pathways (Tu et al., 2009, 2010, 2013; Vincent et al., 2011; Liu et al., 2016; Wei et al., 2016a), investigating features of thalamus-related pain-modulation that may further elucidate the central pain pathway responsible for the manifestation of menstrual pain.

Brain-derived neurotrophic factor (BDNF) is an important mediator of sensory neurotransmission in nociceptive pathways both at spinal and supraspinal levels in humans and animals (Bennett, 2001; Chao et al., 2006; Pezet and McMahon, 2006; Merighi et al., 2008; Maletic and Raison, 2009). Animal studies have confirmed neuronal expression of BDNF in thalamus and other brain regions that are mainly involving nociceptive neurotransmission (Merighi et al., 2008). In human, increased serum BDNF has been consistently reported in chronic pain conditions such as fibromyalgia (Laske et al., 2007; Zanette et al., 2014), chronic musculoskeletal pain (Caumo et al., 2016), chronic tension-type headache and myofascial pain syndrome (Deitos et al., 2015). Higher serum levels of BDNF may be associated with changes in pain modulatory system function. For example, increased levels of BDNF significantly correlated with lower efficiency of pain inhibition in the setting of chronic musculoskeletal pain (Caumo et al., 2016). In postherpetic neuralgia, reduced perceived pain intensity correlated with elevated serum BDNF levels after treatment (Saxena et al., 2016). Although preliminary evidence suggests a significant correlation between central pain modulation and BDNF gene polymorphism in the setting of PDM (Wei et al., 2016b), whether a similar association between pain pathway and serum BDNF is present has not yet been determined.

Here, we aimed to investigate functional connectivity in thalamus-related brain circuits during the period of time without painful menstruation. According to the Human Brainnetome Atlas (a parcellation based on functional and anatomical connectivity), thalamus can be subdivided into 8 subregions through an anatomical connectivity-based parcellation approach (Fan et al., 2016). Venous blood was collected from both PDM subjects and healthy controls (HCs) to determine whether there was a between-group difference in serum BDNF levels. The visual analog scale (VAS) was used to assess menstrual pain intensity when subjects experienced ongoing menstruation. We hypothesized that altered thalamic connectivity would be apparent across the prefrontal cortical areas and sensorimotor cortex due to its widespread connections with cortical regions. We were particularly interested in determining whether specific thalamo-cortical connectivity is correlated with serum BDNF alteration.

## Materials and Methods

Our protocol was approved by the Institutional Ethical Committee of the First Affiliated Hospital of Xi'an Jiaotong University (application No. V1.1) and experimentation was conducted in accordance with the Declaration of Helsinki. All subjects were fully informed of our experimental procedures, and written, informed consent was obtained from each participant.

### Participants

We recruited study subjects from local colleges or universities by advertisement and word of mouth. Potential subjects were screened by the same professional gynecologist for diagnosis; conventional magnetic resonance imaging (MRI) was used to exclude anatomical brain or pelvic abnormalities. Criteria for study inclusion were menstrual cycle regularity (i.e., 27–32 days), subject age of 18–30 years, and subject right-handedness. The VAS (0 = lack of pain; 10 = worst imaginable pain) was used to assess average perceived intensity of menstrual pain over the past 6 months; participants were divided into a PDM group (VAS ≥ 4) and a healthy control group (VAS ≤ 2). Subject exclusion criteria were: (1) presence of organic brain/pelvic conditions (as revealed on MRI); (2) use of medication within 6 months of MRI including hormonal supplements, Chinese medicine or any other drugs affecting the central nervous system; (3) a history of chronic illness, neurological disease or psychiatric disorders, and left handedness; (4) having a metal/pacemaker implant; (5) being pregnant or immediately planning pregnancy; (6) alcohol or drug abuse; and (7) claustrophobia. Consumption of analgesics was not permitted within 24 h prior to the study. MRI was performed during the periovulatory (POV) phase (i.e., on days 12–16 of the menstrual cycle of subjects). Urine kits that measured the surge of luteinizing hormone (indicating impending ovulation) were used for cycle timing.

### Psychological Assessment

The self-rating anxiety scale (SAS) and self-rating depression scale (SDS) were used to evaluate if subjects were anxious or depressed prior to MRI. Participants who had SAS and SDS scores of >50 were not included. The VAS was used to evaluate uterine pain intensity of both women with PDM and HCs. Subjects were asked to report their pain on a scale of 1–10 with 1 signifying a lack of pain and 10 signifying the worst pain imaginable. Every participant was required to report their pain level during the POV phase (VAS-POV). PDM subjects were required to complete the VAS during their next menstrual phase when they felt the worst pain (VAS-MENS; obtained by a follow-up telephone call).

### Serum BDNF Measurement

Venous blood samples were collected in the morning (8:00 AM) from all subjects who were asked to fast overnight (for at least 8 h) at their periovulation, the day they were performing MRI scanning. Blood samples were centrifuged for 10 min at 4,500 rpm at 4°C. Serum was stored at −80°C for assay. Serum BDNF concentrations (CusaBio CSB-E04501h) were assessed by enzyme-linked immunosorbent assay kits which had a lower detection limit of 0.063 ng/ml.

### Imaging Acquisition

Subjects were scanned at the Department of Medical Imaging of First Affiliated Hospital of Xi'an Jiaotong University during their POV phase and were instructed to keep their eyes closed while remaining awake. Subjects were also instructed to not move their head and remain relaxed during scanning. Resting-state functional MRI (rsfMRI) data were acquired using a 3.0-Tesla MRI machine (GE SIGNA HDxt, Milwaukee, WI, USA) with an 8-channel phase array head coil. High-resolution T1-weighted 3D structural images were acquired using an axial fast spoiled gradient recalled sequence with the following parameters: TR/TE = 1,900 ms/2.6 ms; flip angle = 12°; voxel size = 1 mm^3^; data matrix = 256 × 256; field of view (FOV) = 256 × 256 mm; slices = 140. Blood oxygen level-dependent (BOLD) functional images were obtained by means of a T2^*^-weighted single-shot gradient echo-planar-imaging (EPI) sequence with the following parameters: TR/TE = 2,000/30 ms; flip angle = 90°; data matrix = 64 × 64; FOV = 240 × 240 mm; 30 contiguous slices 5 mm thick. A total of 180 functional volumes were acquired. The first 5 EPI scans of each rsfMRI series were discarded for purposes of signal saturation and magnetic field stabilization. After the scan, subjects were asked whether they felt uncomfortable and remained awake during scanning.

### Image Processing

All preprocessing was performed using the Data Processing Assistant for Resting-State fMRI [DPASF (Yan and Zang, 2010); http://www.restfmri.net], which is based on statistical parametric mapping (SPM8; http://www.fil.ion.ucl.ac.uk/spm). The first 10 volumes were discarded for the purpose of data equilibration. Remaining images were slice-time corrected and realigned for head-motion correction. Subjects who had head motion in any direction of >1.5 mm or head rotation 1.5° were excluded from the study. Images were subsequently spatially normalized to the Montreal Neurological Institute (MNI) EPI template, re-sliced to 3 × 3 × 3 mm voxels, and smoothed (Gaussian kernel full-width half-maximum, 6 mm). Detrending and band-pass filtering (0.01–0.08 Hz) (Auer, 2008; Zuo et al., 2010) removed higher frequency physiological noise and lower frequency scanner drift. Finally, nuisance covariates including 24 head motion parameters, as well as white matter, cerebrospinal fluid and global mean signals, were regressed out. Residual images were saved for subsequent analyses and the thalamus was chosen as our seed according to the Human Brainnetome Atlas (Fan et al., 2016). Regional rsfMRI time series were extracted for the region of interest (ROI, i.e., the thalamus) by averaging all voxels at each time point.

### Regional of Interest

**Figure 1:**
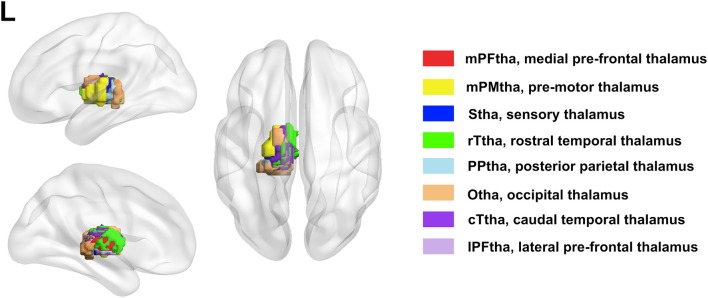
The anatomical location of each thalamus subregion: the medial pre-frontal thalamus (mPFtha), the pre-motor thalamus (mPMtha), the sensory thalamus (Stha), the rostral temporal thalamus (rTtha), the posterior parietal thalamus (PPtha), the occipital thalamus (Otha), the caudal temporal thalamus (cTtha), and the lateral pre-frontal thalamus (IPFtha).

The bilateral thalamus sub-regions were defined using the Brainnetome atlas, which was a parcellation based on structural and functional connectivity. In this atlas, thalamus was symmetrically parceled into 8 subregions in each hemisphere (Figure 1). The thalamus subregions contain the medial pre-frontal thalamus (mPFtha), pre-motor thalamus (mPMtha), sensory thalamus (Stha), rostral temporal thalamus (rTtha), posterior parietal thalamus (PPtha), occipital thalamus (Otha), caudal temporal thalamus (cTtha), lateral pre-frontal thalamus (IPFtha).

### Statistical Analyses

#### Demographic Information, Psychophysiological Measurements, and Serum BDNF Levels

Statistical tests were performed using SPSS Statistics 20.0 (SPSS Inc, Chicago, IL). Data were presented as mean ± SD. A 2-sample *t*-test was applied to evaluate for between-group differences in demographic characteristics and serum BDNF levels. Pearson's correlation analysis was performed between VAS-MENS and serum BDNF levels. Results were considered significant at *p* < 0.05.

### Image Analyses

For each of the left and right thalamic subregions (Figure 1), pearson correlation coefficients were calculated between the mean time series of each seed region and that of each voxel of the whole brain. Two-sample *t*-tests were performed to determine areas with significantly different functional connectivity to the thalamus subregions between two groups, in which age and education were regressed out as covariates (*p* < 0.05, FDR corrected). Multiple comparisons were corrected by a false discovery rate (FDR) of *p* < 0.05. Given the interesting results regarding BDNF level, between-group differences in correlation between thalamic-subregions related functional connectivity and serum BDNF level were performed by using the regression analysis in the PDM and HC groups (a value of *p* < 0.05 was considered significant with FDR corrected).

### Head-Motion Analysis

Correlations in resting-state functional connectivity may be confounded by head motion despite compensatory spatial registration and regression of motion estimates from data (Power et al., 2012). Framewise displacement (FD) is a measure that compares movement from one volume to the next and adds the absolute values of differential realignment estimates at every time point (Power et al., 2012). Absolute displacement (AD) is calculated separately for translation (sum of absolute X, Y, and Z value estimates for a given volume) and rotation (sum of absolute displacement values in pitch, yaw, and roll) (Power et al., 2014). Use of these two measures in our data revealed no significant differences in head motion among our two groups.

## Results

### Demographic Data and Clinical Characteristics

A total of 65 young females were included in this study (36 PDM subjects (age: 24.58 ± 2.59 years, education: 17.22 ± 2.44 years) and 29 age-, education- and gender-matched HCs (age: 24.24 ± 1.92 years, education: 17.1 ± 1.86 years). Participants who had abnormal SAS and SDS scores were excluded. PDM females had significantly higher VAS scores (7.45 ± 0.43) than controls (0.54 ± 0.17) during the menstrual phase (*p* < 0.05). No differences in VAS scores were found during periovulation between the two groups since all subjects reported a lack of pain (i.e., 0). PDM subjects were found to have significantly higher levels of BDNF (1.53 ± 1.32 ng/ml) compared with HCs (0.67 ± 0.65 ng/ml) during periovulation (*p* < 0.05, Table 1). Correlation analysis revealed a positive association between VAS scores of the next menstruation period (VAS-MENS) and serum BDNF levels in PDM subjects; *r* = 0.424, *p* < 0.05.

**Table 1:** Demographic and clinical characteristics at periovulation.

**Items**	**PDM (*n* = 36)**	**HC (*n* = 29)**	***P*-value**
Age (years)	24.58 ± 2.59	24.24 ± 1.92	0.710
Education (years)	17.22 ± 2.44	17.1 ± 1.86	0.252
Duration of menstrual pain (years)	8.6 ± 0.73	–	–
VAS-POV	0	0	–
VAS-MENS	7.45 ± 0.43	0.54 ± 0.17	*p* < 0.05
BDNF (ng/m)	1.53 ± 1.32	0.67 ± 0.65	*p* < 0.05
SAS	34.62 ± 1.36	32.14 ± 1.20	0.183
SDS	43.25 ± 1.70	40.23 ± 1.53	0.197

*PDM, primary dysmenorrhea; HC, healthy controls; VAS, visual analog scale; POV, periovulation; MENS, menstruation; BDNF, brain-derived neurotrophic factor; SAS, self-anxiety scale; SDS, self-depression scale*.

### Functional Connectivity Analysis of Thalamus Subregions During the POV Phase

Data were checked using both FD and AD (as mentioned above); no significant differences in head motion were found among the two groups.

**Figure 2:**
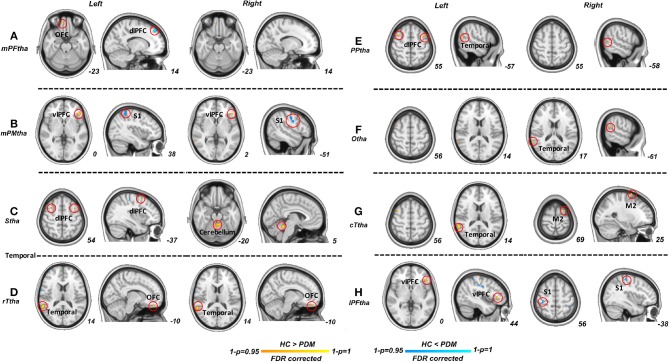
Significant functional connectivities for each thalamus subregions between PDM and HCs. Compared with HCs, significant higher FC (blue) of thalamic subregions were mainly related to dlPFC, S1; significant lower FC (red) of thalamic subregions were maliny related to OFC, vlPFC, dlPFC, M2, temporal cortex and cerebellum (*p* < 0.05; FDR corrected). PDM, primary dysmenorrhea; HC, healthy controls; FC, functional connectivity; dlPFC, dorsal lateral prefrontal cortex; SI, primary sensory cortex; M2, supplementary motor cortex; OFC, orbitofrontal cortex; vlPFC, ventral lateral prefrontal cortex.

Significant differences were found in resting-state functional connectivities of thalamus subregions between PDM and HCs (Figure 2). As presented in Figure 2, compared with HCs, the left medial prefrontal thalamus showed higher FC between orsal lateral prefrontal cortex (dlPFC, Brodmann areas BA9) and lower FC between orbitofrontal cortex (OFC, BA11); the pre-motor thalamus showed higher FC between primary somatosensory cortex (S1, BA2) and lower FC between ventral lateral prefrontal cortex (vlPFC, BA47); the sensory thalamus showed lower FC between bilateral dlPFC and cerebellum; the rostral temporal thalamus showed lower FC between OFC and temporal cortex (BA21, 22); the posterior parietal thalamus showed lower FC between dlPFC and temporal cortex; the right occipital thalamus showed lower FC between temporal cortex; the caudal temporal thalamus showed lower FC between temporal cortex and supplementary motor cortex (M2); the lateral pre-frontal thalamus showed higher FC between S1 and lower FC between vlPFC (*p* < 0.05; FDR corrected). No significant difference was found in ROI of right medial pre-frontal thalamus and left occipital thalamus. Detailed information of FC between thalamic-subregions and other brain regions was shown in Table 2.

**Table 2:** Between-group differences in FC of the thalamic subregions (HC vs PDM, *p* < 0.05, FDR corrected).

	**Brain region**	**Side**	**Voxels**	**Voxels with maximum effect**				**Brain region**	**Side**	**voxels**	**Voxels with maximum effect**			
				**MNI**			***t*-value**				**MNI**			***t*-value**
				**x**	**y**	**z**					**x**	**y**	**z**	
(a)	L-medial pre-frontal thalamus							R-medial pre-frontal thalamus						
	Frontal Superior Orbital	R	12	15	42	−24	5.14	No						
	Frontal Superior	L	13	−12	42	45	−5.18							
	Rolandic Operculum	R	11	66	−3	12	−5.06							
(b)	L-pre-motor thalamus							R-pre-motor thalamus						
	Frontal Inferior Orbital	L	19	−42	30	−3	6.35	Postcentral	R	79	45	−15	39	−5.37
	Parietal Superior	L	14	−39	−45	57	−5.02	Frontal Inferior	L	10	−42	30	0	5.14
								Temporal Mid	R	10	63	−36	6	5.55
								Calcarine	L	17	−15	−72	18	−5.35
								Postcentral	L	17	−42	−18	33	−5.07
								Parietal Inferior	L	23	−36	−45	48	−5.12
(c)	L-sensory thalamus							R-sensory thalamus						
	Frontal Middle	R	16	33	9	54	5.26	Cerebellum	L	42	−3	−48	−21	6.29
	Frontal Middle	L	14	−36	6	54	5.15	Temporal Middle	R	11	63	−36	−6	5.48
(d)	L-rostral temporal thalamus							R-rostral temporal thalamus						
	Frontal Superior Orbital	R	11	15	45	−24	5.21	Frontal Superior Orbital	R	10	15	45	−24	5.42
	Temporal Middle	R	10	63	−48	12	5.23	Temporal Middle	R	10	63	−48	12	5.24
(e)	L-posterior parietal thalamus							R-posterior parietal thalamus						
	Temporal Middle	R	19	60	−57	12	4.87	Occipital Inferior	L	50	−21	−99	−9	−5.86
	Frontal Middle	R	10	30	6	54	4.87	Occipital Inferior	R	18	33	−90	−6	−5.53
	Frontal Middle	L	10	39	6	54	4.87							
	Vermis	R	15	3	−57	−21	5.05							
	Frontal Inferior Orbital	L	10	42	30	−3	5.21							
(f)	L-occipital thalamus							R-occipital thalamus						
	No							Temporal Middle	R	10	66	−48	15	5.17
								Occipital Inferior	L	22	−21	−99	−9	−4.98
(g)	L-caudal temporal thalamus							R-caudal temporal thalamus						
	Temporal Mid	R	33	63	−48	12	5.7	Occipital Inferior	R	20	30	−96	−3	−5.55
								Frontal Superior	L	13	−24	3	69	5.64
(h)	L-lateral pre-frontal thalamus							R–lateral pre–frontal thalamus						
	Frontal Inferior Orbital	L	15	−42	30	−3	5.86	Postcentral	R	12	39	−30	54	−5.04
	Parietal Inferior	L	15	−39	−45	54	−5.21	Occipital Inferior	L	19	−24	−99	−12	−5.24
								Occipital Inferior	R	12	33	−90	−6	−5.16

*FC, functional connectivity; HC, healthy control; PDM, primary dysmenrorhea; L, left; R, right; FDR, false discovery rate*.

*Peak coordinates refer to the MNI space; MNI, Montreal Neurological Institute*.

*Difference reported is t-value from a two-sample t-text*.

### Correlation Analyses Between Functional Connectivity and Serum BDNF Levels

**Figure 3:**
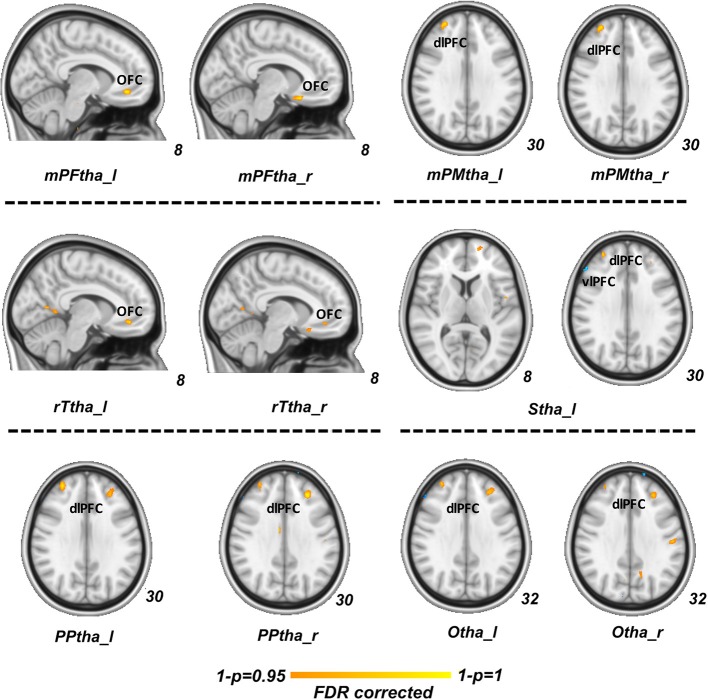
Regression analysis between group difference in correlation of thalamic-subregions related FC and serum BDNF level. Regions of vlPFC (PDM>HCs, blue), OFC (PDM < HCs, red) and dlPFC (PDM < HCs, red) showed a significant interaction effect between two groups (*p* < 0.05, FDR corrected). FC, functional connectivity; BDNF, brain-derived neurotrophic factor; vlPFCM, ventral lateral prefrontal cortex; PDM, primary dysmenorrhea; HC, healthy controls; OFC, orbitofrontal cortex; dlPFC, dorsal lateral prefrontal cortex.

In our regression analysis, the vlPFC (PDM>HCs), OFC (PDM < HCs) and dlPFC (PDM < HCs) showed a significant interaction effect with serum BDNF level between two groups (*p* < 0.05, FDR corrected, Figure 3).

## Discussion

PDM is characterized by spontaneous pain experienced at the onset of menstruation but generally absent otherwise. Either physiological or neuroimaging studies have demonstrated hypersensitivity to exogenous nociceptive stimuli (hyperalgesia) during the periovulatory period, likely due to dysfunction of central pain perception in the setting of this condition (Iacovides et al., 2015). The purpose of this study was to explore functional connectivity changes of thalamic subregions in patients with PDM. According to a priori subregion atlas of the thalamus, we found that PDM patients had significant higher or lower functional connectivity between different thalamus subregions and cortical brain regions than HCs. We also evaluated differences in serum BDNF levels and found them to be significantly higher in PDM during periovulation. After adjusting for relevant confounders, the vlPFC, OFC, and dlPFC showed a significant interaction effect between two groups. This study provided novel evidence of the key role of the thalamus in pathophysiological mechanism of PDM and abnormal connectivity patterns of the thalamic subregions.

### Abnormal Functional Connectivity Between Thalamus Subregions and Pain Perception or Modulation Circuits

The thalamus is located in the central forebrain and serves a crucial role in top-down pain processing (Gauriau and Bernard, 2004; EG, 2007a,b; Todd, 2010) and cortico-cortical communication (Sherman, 2016). Peripheral nociceptive information is mainly transmitted through the spinothalamic tract to the medial thalamus, within which the ventrobasal complex is mostly associated with sensory and discriminative roles, whereas the intralaminar nuclei/posterior complex are involved with motivational, emotional and reactive aspects of pain (EG, 2007a,b; Todd, 2010). In humans, thalamic activation has been consistently observed in studies concerning endogenous and exogenous nociceptive stimuli (Helmchen et al., 2006; Friebel et al., 2011). In particular, spatial aspects of pain were reported to be preferentially conveyed through the anterior and medial thalamus to the posterior parietal and dorsolateral prefrontal cortices (Oshiro et al., 2007), whereas pain intensity discrimination significantly activated the ventral stream of brain regions, such as the medial thalamus, anterior insula and ventromedial prefrontal cortex (Oshiro et al., 2009). These studies further confirmed the critical role of the thalamus in pain transmission and pain intensity coding (Bingel and Tracey, 2008; Schmid et al., 2015).

In the current studies, the prefrontal thalamus (mPFtha, IPFtha) showed higher functional connectivities between, S1, dlPFC and lower functional connectivities between OFC and vlPFC. In contrast to the prefrontal thalamus, the motor and sensory thalamic subregions (mPMtha, Stha) showed higher functional connectivity between S1, but lower functional connectivity with vlPFC and dlPFC. The SI cortex has been postulated to play a predominant role in the perception of pain (localization and discrimination). However, it is now indicated that plasticity of SI is one of the causes in meditating or sustaining chronic pain, not merely a simple and passive epiphenomenon following tissue or nerve injury as previous reported (Kim et al., 2017). For example, hyperexcitability and reorganization of SI cortex have been reported to serve active roles in the chronicity of neuropathic pain (Seifert and Maihofner, 2009). Findings from EEG researches have shown that, sensorimotor cortical activity in the chronic stage of pain is typically characterized by greater S1 and M1 activity (Diers et al., 2007; Te et al., 2017), which might reflect a maladaptive neuroplasticity and the adoption of simplified movement strategies in the transition to sustained pain (Schabrun et al., 2016). Furthermore, the neural activity in brain areas associated with pain signal processing, including thalamus, has been reported to be modulated by the manipulation of the SI cortex (Eto et al., 2011). We therefore speculated that the higher communication between thalamic subregions and sensory cortex in our results might indicate a similar maladaptive or adoptive alteration in patients associated with long-term menstrual pain.

The thalamus mediates communication between multiple functional networks (Sherman, 2016) involving cognitive and affective regulation such as pain-related attention, emotion, memory formulation, and inhibition (Peyron et al., 1999; Mathur et al., 2015). Among thalamus-related pain pathways, the dlPFC has been identified as a critical region in nociceptive processing and pain modulation, mostly involving the pathway of descending inhibition (Wolff et al., 2015). During thermal stimulation, dynamic activity of the dlPFC was found to negatively correlate to activity among the midbrain and thalamus, as well as the intensity of perceived pain, suggesting a top-down inhibitory neural circuit along the thalamus-related ascending pathway that is modulated by descending fibers from the dlPFC (Lorenz et al., 2003). In a study by Sevel et al., individuals with stronger thalamic-dlPFC connections tended to exhibit greater responses to analgesia via effective dlPFC-derived descending pain modulation (Sevel et al., 2015). There is also converging evidence that the dlPFC has an important role in cognitive components of the pain experience (Seminowicz and Moayedi, 2017). Therefore, findings of higher or lower functional connectivity of dlPFC as mentioned above may represent functional alterations within the pain modulation circuit. It is noteworthy that cognitive control effect in pain relief has in part been attributed to brain network comprising prefrontal regions including the dlPFC, OFC, and vlPFC (Bingel et al., 2007). The OFC receives information from the medial dorsal nucleus of the thalamus and is thought to reflect emotion and reward in decision making (Rolls, 2000; Rempel-Clower, 2007). It was also reported to be involved in the process of pleasure-induced pain inhibition (Becker et al., 2017). The vlPFC, although not being fully investigated in pain conditions, is thought to play a critical role in motor inhibition and cognitive control (Levy and Wagner, 2011). In addition, lower functional connectivity of dlPFC could be also found in other thalamic subregions including PPtha in our PDM patients. According to these, findings of higher/lower functional connectivity between these prefrontal cortex and thalamic subregions may suggest a widespread alteration in maladaptive modulation of pain perception and inhibition during the periovulatory period even in the absence of menstrual pain. Furthermore, investigations need to be performed to clarify the function of the prefrontal cortex in the chronicity of this repetitive pelvic pain.

Another interesting finding was that PDM females had lower functional connectivity between the temporal cortex and regions of rTtha, PPtha, Otha, and cTtha than HCs. Previous researches have demonstrated the important role of temporal cortex in auditory, visual, memory, or emotion processing (Anderson et al., 2017; Marshall et al., 2019), but few studies focus on the underlying effect in nociceptive information transmission. Recently, a neuroimaging study implicated that the medial temporal lobe may involve in chronic low back pain patients. However, the evidence of the link between temporal cortex and chronic pain remains elusive and more research is needed to reveal the abnormal thalamo-temporal connectivity in our results.

### Interaction Between Serum BDNF and Thalamic-Subregions Related Functional Connectivity

BDNF is produced in the central nervous system and has been proposed to be a marker of neural plasticity (Deitos et al., 2015). Although human and animal studies have suggested an important role of BDNF in mediating sensory neurotransmission within pain circuitry (Merighi et al., 2008; Thibault et al., 2014), whether BDNF is associated with increased or decreased nociceptive effects remains unclear. Here, we have found higher serum BDNF levels in PDM subjects as compared to healthy controls. We further found a positive correlation between serum BDNF levels and MENS-VAS, inferring that higher serum BDNF levels might correlate with greater perceived menstrual pain. Our reasoning is corroborated by recent studies evaluating chronic pain conditions as well as animal experiments. A recent animal study reported enhanced overexpression of BDNF to be associated with nociceptive activity in the medial thalamus, considered a key factor in central post-stroke pain (Shih et al., 2017). A study of cortical excitability using transcranial magnetic stimulation in healthy males revealed higher serum BDNF levels to correlate with thermal pain hypersensitivity and reduced pain inhibition during noxious heterotopic stimulation (Dussan-Sarria et al., 2017). Additionally, greater BDNF levels were shown to be inversely correlated with disinhibition of cortical excitation and numerical pain scaling in patients with chronic musculoskeletal pain, suggesting that higher serum BDNF levels may be involved in the dysfunction of descending inhibitory pain modulation (Caumo et al., 2016). Higher serum BDNF levels have also been reported in the setting of other chronic pain conditions, such as fibromyalgia (Laske et al., 2007; Zanette et al., 2014), chronic musculoskeletal pain (Caumo et al., 2016), migraine, and chronic tension-type headache (Tanure et al., 2010; Deitos et al., 2015). These findings strongly implicate that higher serum BDNF levels may associate with the physiopathology of chronic menstrual pain and other chronic pain.

Another interesting finding of our research is that between group difference functional connectivity of vlPFC, OFC and dlPFC with thalamic-subregions showed a significant interactive effect with serum BDNF level. As is well discussed above, the vlPFC is thought to play a critical role in motor inhibition and cognitive control (Levy and Wagner, 2011); the OFC is thought to underpin the evaluation of aversive stimuli and regulation of negative emotion (Kringelbach, 2005); the dlPFC is identified as a critical region in nociceptive processing and pain modulation, mostly involving the pathway of descending inhibition (Wolff et al., 2015). These regions have been reported to participate in brain cognitive control effect in pain relief (Bingel et al., 2007). The interactive effect between serum of BDNF level and thalamic-subregions seeded functional connectivity with the prefrontal cortex may indicate a modulative role of BDNF in brain mechanism along with chronic menstrual pain. However, there hasn't been sufficient evidence to suggest the relationship between BDNF and brain alteration in chronic menstrual pain. Only a few studies have shown that BDNF levels are altered in patients suffering menstrual pain and that BDNF gene polymorphisms are related with genetic susceptibility to PDM (Lee et al., 2014; Wei et al., 2016b). Through the screening and genotyping of 99 women suffering PDM and 101 healthy females, *BDNF* Met/Met homozygosity was revealed to be associated with an increased risk of developing PDM (Lee et al., 2014). The *BDNF* Val66Met polymorphism was further suggested to be associated with the dynamics of functional connectivity involved in descending pain modulation in PDM (Wei et al., 2016b). A comparison of blood, plasma, and brain tissue from different animal species revealed that blood and plasma BDNF levels accurately reflect those of brain tissue (Klein et al., 2011). Accordingly, higher serum BDNF levels or the *BDNF* Val66Met polymorphism in PDM reflect abnormal BDNF secretion in brain tissue, likely a critical factor in the pathophysiology of this condition. These evidences strongly suggested an important modulative role of BDNF in development and chronicity of central nervous system dysfunction. According to the above research and our findings, we can postulate that BDNF may play an important role in central pain perception or modulation pathway along with long-term menstrual pain. Further studies are required to investigate how BDNF acts in modulation of PDM pain.

## Limitations

Here, we merely analyzed women at periovulation (a time without ongoing menstrual pain) using neuroimaging. We could not identify whether thalamus-related functional connectivity was normal or disrupted in response to cyclic external stimuli; neither could we determine whether serum BDNF levels were higher in women with PDM primarily due to chronic menstrual pain. Casual-relation and longitudinal research is required to determine the correlation of cerebral abnormality characteristics and serum BDNF levels in women suffering chronic menstrual pain. Future research will provide greater in-depth understanding of this condition and eventually lead to effective clinical management strategies.

## Conclusions

In our study, abnormal functional connectivity between the thalamic-subregions and other brain regions involved in pain perception and modulation was revealed during the periovulatory phase in women with chronic PDM. Furthermore, significantly higher serum BDNF levels were found in PDM subjects when compared with healthy controls during periovulation. Higher serum BDNF levels correlated with greater pain intensity reported during menstruation. In addition, the functional connectivity of thalamic-subregions showed a significant interactive effect correlated with serum BDNF level between the two groups, suggesting that BDNF plays a role in the development and chronicity of central nervous system dysfunction in the setting of chronic menstrual pain. Our findings revealed greater dysfunction in thalamus-related functional connectivity even in the absence of ongoing menstrual pain that also closely correlated with serum BDNF levels. Such alterations provided more accurate information about the involvement of the thalamus in the pathophysiology of PDM.

## Author Contributions

WD and MZ were responsible for the study concept and design. FH, HL, and KW contributed to MRI data acquisition. JY, LY, and JL performed data analysis and interpreted findings. WD and FH drafted the manuscript and approved the final draft for submission. All authors reviewed the paper.

### Conflict of Interest

The authors declare that the research was conducted in the absence of any commercial or financial relationships that could be construed as a potential conflict of interest.
